# *Porphyromonas gingivalis* lipopolysaccharide (Pg-LPS) influences adipocytes injuries through triggering XBP1 and activating mitochondria-mediated apoptosis

**DOI:** 10.1080/21623945.2020.1856527

**Published:** 2021-01-04

**Authors:** Ying-Tao Lv, Jin-Jin Zeng, Jia-Yi Lu, Xue-Yang Zhang, Ping-Ping Xu, Yuan Su

**Affiliations:** a Department of Oral and Maxillofacial Surgery, Stomatological Hospital, Southern Medical University, Guangzhou, China; b Department of Periodontology, Stomatological Hospital, Southern Medical University, Guangzhou, China; c, Stomatology Center, Shunde Hospital, Southern Medical University (The First People’s Hospital of Shunde), Shunde, China

**Keywords:** XBP1, adipocytes, Pg-LPS, inflammation, apoptosis

## Abstract

Obesity is an important public-health problem worldwide. This study aimed to determine effects of porphyromonas gingivalis lipopolysaccharide (Pg-LPS) on adipocytes injuries and explore associated mechanisms. Adipocytes were isolated from SD rats. pLVX-XBP1 (XBP1 over-expression) and pLVX-XBP1-RNAi (silencing XBP1) were structured and transfected into adipocytes. All adipocytes were divided into pLVX-NC, pLVX-XBP1, pLVX-NC+Pg-LPS and pLVX-XBP1+ Pg-LPS group. Oil-Red O staining was employed to identify isolated adipocytes. Quantitative real-time PCR (qRT-PCR) was used to examine gene transcription of IL-6, TNF-α, leptin, adiponectin. Western blotting was used to detect Bax and caspase-3 expression. Adipocytes were successfully isolated and identified with Oil-Red O staining. Both XBP1 mimic and XBP1 RNAi were effectively transfected into adipocytes with higher expressing efficacy. XBP1 over-expression significantly aggravated Pg-LPS induced inflammatory response compared to adipocytes without Pg-LPS treatment (p<0.05). Pg-LPS significantly enhanced leptin and inhibited adiponectin expression by up-regulating XBP1 expression (p<0.05). XBP1 silence significantly alleviated Pg-LPS induced inflammatory response and reduced leptin, enhanced adiponectin expression in Pg-LPS treated adipocytes compared to adipocytes without Pg-LPS treatment (p<0.05). Pg-LPS induced apoptosis of adipocytes by enhancing XBP1 expression and modulating Bcl-2/Bax pathway associated molecules. In conclusion, Porphyromonas gingivalis lipopolysaccharide (Pg-LPS) induces adipocytes injuries through modulating XBP1 expression and initialling mitochondria-mediated apoptosis.

## Introduction

In recent years, obesity has been become an important public-health problem affecting billion peoples in the whole world [1,2]. Obesity is considered to be a risk factor for many disorders, including cardiovascular disorders, type 2 diabetes mellitus (T2DM), insulin resistance, metabolic syndrome and cancers [3]. Therefore, it’s critical for understanding molecular mechanism underlying fat expansion and physiological functions of adipose tissues [4]. Adipose tissues are mainly composed of various cell populations, especially for adipocytes and non-adipocytes [5]. Adipocytes contribute to fat accumulation and non-adipocytes are derived post-collagenase digestion. A previous study [6] reported that inflammation and death of adipocytes in adipose tissues were associated with obesity. Therefore, it’s critical to discover the therapeutic targets and clarify the associated mechanism of inflammation or apoptosis of adipocytes in adipose tissues.

*Porphyromonas gingivalis* (*P. gingivalis*) is a kind of Gram-negative (G^−^) anaerobic bacterium and considered to be a major pathogen causing chronic inflammation in periodontal disorders [7]. The lipopolysaccharide (Pg-LPS) located on surface of *P. gingivalis* could interact with toll-like receptors and further trigger the cytokines and chemokines release [8]. A former study [9] also reported that Pg-LPS could promote production of interleukins (ILs) and tumour necrosis factors (TNFs) through activating TLR4-associated signalling pathways. Our previous study [10] also reported that the periodontitis could induce inflammation of the adipose tissues. Therefore, in this study, we employed the Pg-LPS to stimulate inflammatory adipocytes and observed the effects of Pg-LPS on adipocytes injuries.

Adipogenesis is a differentiated process that generates the lipid-laden mature adipocytes deriving from fibroblast-like pre-adipocytes, and about 100 molecules and their associated pathways have been proven to participate in this process [11]. Among the adipogenesis associated signalling pathways, the most widely investigated one is the inositol requiring enzyme 1 (IRE1)-X-box binding protein 1 (XBP1) signalling pathway. The previous studies [12–14] reported that XBP1 plays critical roles in a series processes in cells, such as cell apoptosis, cell differentiation, liver lipogenesis and modulation for immune response. However, whether XBP1 involves in accumulation of adipocytes and causes adipocytes injuries have never been clarified.

Therefore, this study aimed to investigate the effects of *porphyromonas gingivalis* lipopolysaccharide (Pg-LPS) on adipocytes injuries and explored the associated mechanisms. Also, we explored whether XBP1 modulate the inflammation and apoptosis of adipocytes undergoing the Pg-LPS stimuli.

## Results

### Adipocytes were successfully isolated and identified

**Figure 1.**
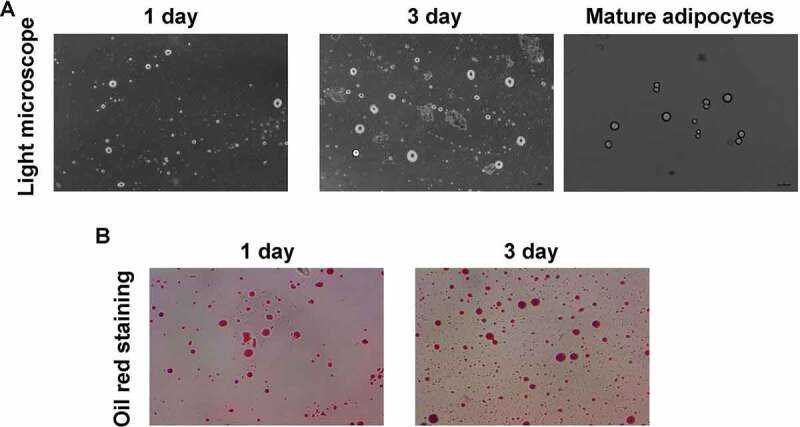
Evaluation for the isolation and identification for adipocytes using light microscope and Oil Red O staining, respectively. (a). Light microscope image. (b). Oil Red O staining for identifying the adipocytes

**Figure 2.**
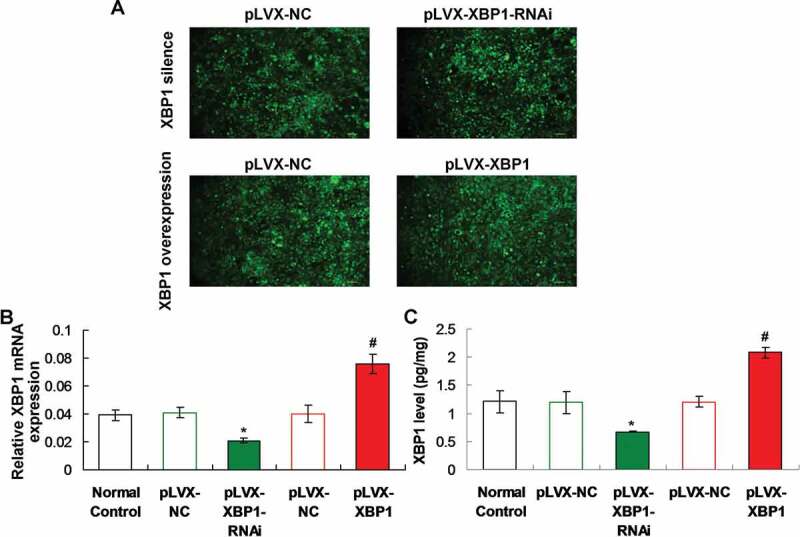
Determination for the pLVX-XBP1 and pLVX-XBP1-RNAi transfection in the adipocytes using fluorescence assay, qRT-PCR and ELISA. (a). Fluorescence assay for determining transfection of pLVX-XBP1 and pLVX-XBP1-RNAi transfection. (b). qRT-PCR assay for determining expressive efficacy of pLVX-XBP1 and pLVX-XBP1-RNAi. (c). ELISA for determining expressive efficacy of pLVX-XBP1 and pLVX-XBP1-RNAi. * *p* < 0.05 *vs*. pLVX-NC group, ^#^ *p* < 0.05 *vs*. pLVX-XBP1 group

As the light microscope image illustated (Figure 1(a)), adipocytes were significantly isolated and cultured for 1 day and 3 days, respectively. The mature adipocytes were obtained (Figure 2(a)), which demonstrated typical morphology. Also, the Oil Red staining results illustrated that the isolated cells were positive stained with Oil Red O (Figure 1(b)), and the cells were amplified following with the culture time (from 1 to 3 days). These results showed that the isolated cells were the adipocytes.

### Both XBP1 mimic and XBP1 RNAi demonstrated higher efficacy

The fluorescence images showed that the pLVX-NC, pLVX-XBP1-RNAi and pLVX-XBP1 were expressed with higher efficacy (Figure 2(a)). Both of qRT-PCR assay and ELISA results also indicated that XBP1 gene transcriptions (Figure 2(b)) or XBP1 levels (Figure 2(c)) were lower significantly in pLVX-XBP1-RNAi group compared to those in pLVX-NC group (*p* < 0.05). Moreover, the XBP1 gene transcriptions (Figure 2(b)) or XBP1 levels (Figure 2(c)) were higher significantly in pLVX-XBP1 group compared to those in pLVX-NC group (*p* < 0.05). Therefore, due to the fluorescence images, qRT-PCR findings and ELISA results, both of XBP1 mimic and XBP1 RNAi were successfully transfected with higher efficacy.

### XBP1 over-expression aggravated Pg-LPS induced inflammatory response in adipocytes

**Figure 3.**
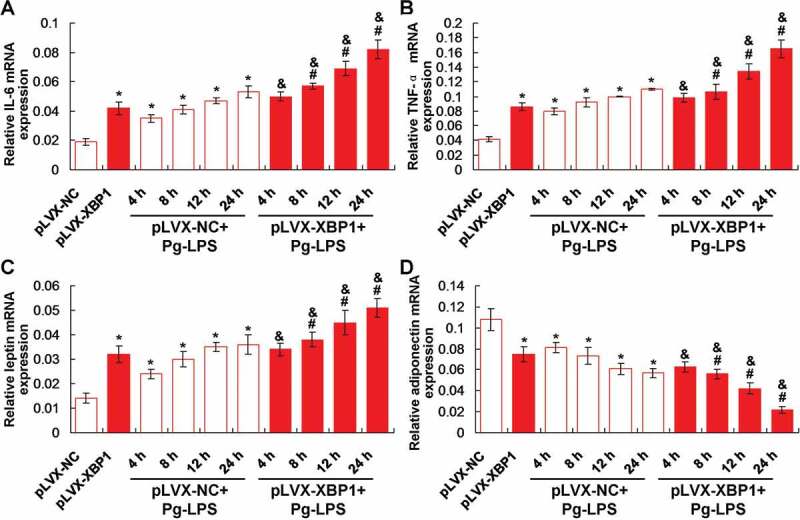
Effects of XBP1 over-expression on the inflammatory response and production of leptin and adiponectin using qRT-PCR assay. (a). Statistical analysis for the IL-6 gene transcription. (b). Statistical analysis for the TNF-α gene transcription. (c). Statistical analysis for the leptin gene transcription. (d). Statistical analysis for the adiponectin gene transcription. * *p* < 0.05 *vs*. pLVX-NC group, ^#^ *p* < 0.05 *vs*. pLVX-XBP1 group, ^&^ *p* < 0.05 *vs*. pLVX-NC+Pg-LPS group

In order to observe effects of XBP1 expression on the Pg-LPS induced inflammation, the inflammatory factors, IL-6 and TNF-α, were examined using qRT-PCR assay. The results showed that XBP1 over-expression (pLVX-XBP1) significantly increased the IL-6 (Figure 3(a)) and TNF-α (Figure 3(b)) gene transcription compared to those in pLVX-NC group (*p* < 0.05). Meanwhile, the Pg-LPS treatment (pLVX-NC+Pg-LPS group) significantly enhanced the IL-6 (Figure 3(a)) and TNF-α (Figure 3(b)) gene transcription compared to those in pLVX-NC group (*p* < 0.05), at 4 h, 8 h, 12 h and 24 h post-treatment. Furthermore, XBP1 over-expression in Pg-LPS treated adipocytes (pLVX-XBP1+ Pg-LPS group) significantly up-regulated the IL-6 (Figure 3(a)) and TNF-α (Figure 3(b)) gene transcription compared to those in adipocytes of pLVX-NC+Pg-LPS group (*p* < 0.05), at 4 h, 8 h, 12 h and 24 h post-treatment, respectively.

### Pg-LPS enhanced leptin and inhibited adiponectin expression by up-regulating XBP1 expression

The findings identified that XBP1 over-expression (pLVX-XBP1) remarkably increased leptin (Figure 3(c)) and decreased adiponectin (Figure 3(d)) gene transcription compared to those in pLVX-NC group (*p* < 0.05). Also, Pg-LPS treatment (pLVX-NC+Pg-LPS group) significantly up-regulated leptin (Figure 3(c)) and down-regulated adiponectin (Figure 3(d)) gene transcription compared to those in pLVX-NC group (*p* < 0.05). Moreover, XBP1 over-expression in Pg-LPS treated adipocytes (pLVX-XBP1+ Pg-LPS group) significantly increased leptin (Figure 3(c)) and decreased adiponectin (Figure 3(d)) gene transcription compared to those in adipocytes of pLVX-NC+Pg-LPS group (*p* < 0.05), at 4 h, 8 h, 12 h and 24 h post-treatment, respectively.

### XBP1 silence alleviated Pg-LPS induced inflammatory response in adipocytes

**Figure 4.**
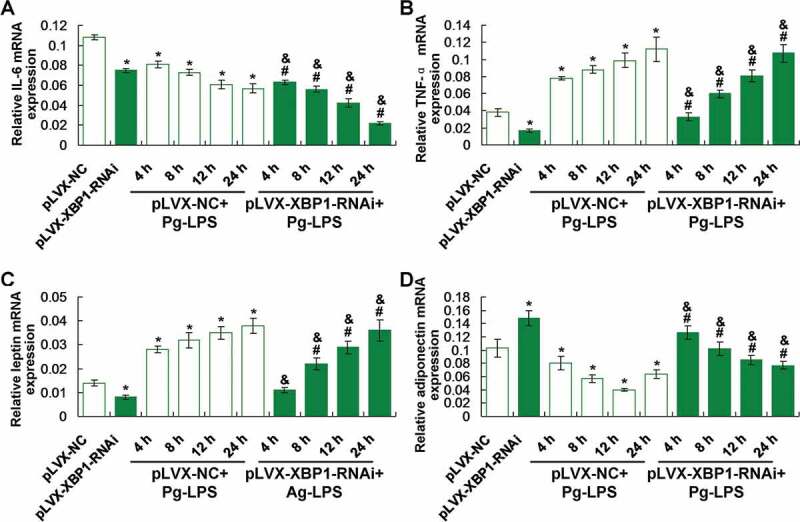
Evaluation for the effects of XBP1 silence on inflammatory response and secretion of leptin and adiponectin using qRT-PCR assay. (a). Statistical analysis for the IL-6 gene transcription. (b). Statistical analysis for the TNF-αgene transcription. (c). Statistical analysis for the leptin gene transcription. (d). Statistical analysis for the adiponectin gene transcription. * *p* < 0.05 *vs*. pLVX-NC group, ^#^ *p* < 0.05 *vs*. pLVX-XBP1 group, ^&^ *p* < 0.05 *vs*. pLVX-NC+Pg-LPS group

XBP1 silence (pLVX-XBP1-RNAi) significantly down-regulated IL-6 (Figure 4(a)) and TNF-α (Figure 4(b)) gene transcription compared to those in pLVX-NC group (*p* < 0.05). The XBP1 silence (pLVX-XBP1-RNAi+Pg-LPS group) significantly down-regulated IL-6 (Figure 4(a))) and TNF-α (Figure 4(b)) gene transcription compared to those in pLVX-NC+Pg-LPS group (*p* < 0.05), at 4 h, 8 h, 12 h and 24 h post treatment, respectively.

### XBP1 silence reduced leptin and enhanced adiponectin expression in Pg-LPS treated adipocytes

Our findings demonstrated that XBP1 silence (pLVX-XBP1-RNAi) significantly decreased leptin (Figure 4(c)) and increased adiponectin (Figure 4(d)) gene transcription compared to those in pLVX-NC group (*p* < 0.05). However, XBP1 silence (pLVX-XBP1-RNAi+Pg-LPS group) significantly reduced leptin (Figure 4(c)) and enhanced adiponectin (Figure 4(d)) gene transcription compared to those in pLVX-NC+Pg-LPS group (*p* < 0.05), at 4 h, 8 h, 12 h and 24 h post-treatment, respectively.

### Pg-LPS induced apoptosis of adipocytes via enhancing XBP1 and modulating Bcl-2/Bax pathway molecules expression

**Figure 5.**
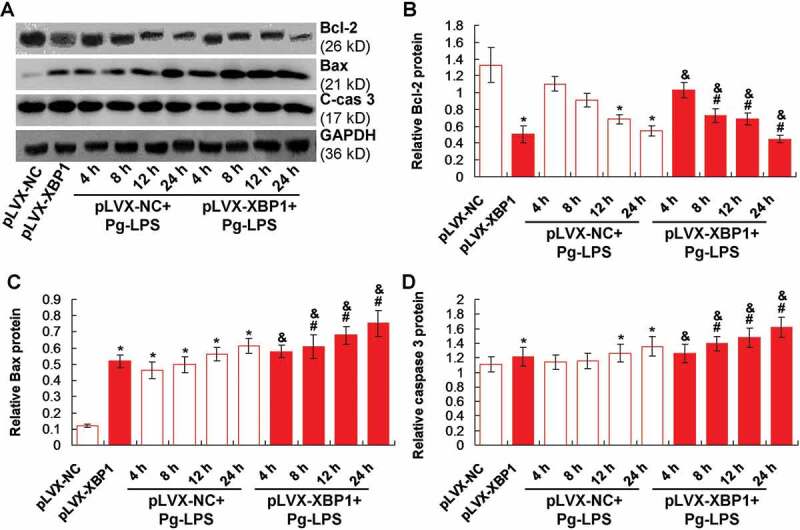
XBP1 over-expression induced Bcl-2 down-regulation and Bax/cleave-caspase 3 (C-cas 3) protein up-regulation. (a). Western blotting image for Bax and caspase 3 expression. B. Statistical analysis for the Bcl-2 expression. (c). Statistical analysis for the Bax expression. (d). Statistical analysis for the caspase 3 expression. * *p* < 0.05 *vs*. pLVX-NC group, ^#^ *p* < 0.05 *vs*. pLVX-XBP1 group, ^&^ *p* < 0.05 *vs*. pLVX-NC+Pg-LPS group

We speculated that the inflammation in adipocytes might be associated with the apoptosis, therefore, we evaluated apoptosis status by examining Bcl-2, Bax and caspase 3 molecule [15] (Figure 5(a)). The results showed that XBP1 over-expression (pLVX-XBP1) remarkably decreased the Bcl-2 expression compared to that in pLVX-NC group (Figure 5(b)), *p* < 0.05). However, the Pg-LPS treatment (pLVX-NC+Pg-LPS group) obviously reduced the Bcl-2 expression compared to that in pLVX-NC group (Figure 5(b), *p* < 0.05).

Moreover, XBP1 over-expression (pLVX-XBP1) significantly enhanced Bax and caspase 3 expression compared to that in pLVX-NC group (*p* < 0.05). Meanwhile, the Pg-LPS treatment (pLVX-NC+Pg-LPS group) obviously induced Bax (Figure 5(c)) and caspase 3 (Figure 5(c)) expression compared to those in pLVX-NC group (*p* < 0.05). Additionally, the XBP1 over-expression in Pg-LPS treated adipocytes (pLVX-XBP1+ Pg-LPS) significantly strengthened the inducible effects of Pg-LPS on the Bax (Figure 5(c)) and caspase 3 (Figure 5(d)) expression compared to those in adipocytes of pLVX-NC-Pg-LPS group (*p* < 0.05). Therefore, the XBP1 over-expression could induce Bax-mediated apoptosis both in blank adipocytes and Pg-LPS treated adipocytes.

## Discussion

XBP1 is an important molecule that regulates the adipogenesis [16]. In this research, we provided the promising evidence that XBP1 could modulate the inflammation and apoptosis of adipocytes. Actually, there are some controversial issues for the adipogenesis both *in vitro* and *in vivo* levels. A few studies [16,17] proved that adipogenesis is usually inhibited by the XBP1 depletion. However, Gregor et al [18]. found that deletion of the XBP1 in adipocytes suppresses the adipogenesis in 3T3-L1 cells. Therefore, the present study attempted to clarify the mechanism of Pg-LPS caused inflammation, apoptosis, and adipocytes injuries.

Here, we established both of XBP1 mimic plasmid (pLVX-XBP1) and XBP1 silence plasmid (pLVX-XBP1-RNAi), both of which were transfected into adipocytes to evaluate associated functions. The isolated cells were identified using the Oil Red O staining method, which is a classical approach for identifying adipocytes [19]. The Oil Red O staining results showed that the isolated cells demonstrated the obvious characteristics of adipocytes, which confirms the application of adipocytes in the following experiments. Then, the expressive efficacy of pLVX-XBP1 and pLVX-XBP1-RNAi transfections were evaluated using fluorescence assay and qRT-PCR assay, respectively. The fluorescence assay demonstrated higher transfection efficiency of the lentivirus and qRT-PCR also exhibited higher expressing efficacy. Therefore, both of pLVX-XBP1 and pLVX-XBP1-RNAi could be reliably applied to display the XBP1 over-expression and XBP1 silence in the following experiments.

The pathological bacteria and sensitive inflammatory stimulus, could activate the cysteine proteinases and then trigger the secretion of pro-inflammatory factors, such as TNF-α, matrix metalloproteinases (MMPs), IL-6, prostaglandins (PGE), proteases and *so on* [20,21]. Pg-LPS, as a product of the *P. gingivalis*, could promote the production and secretion of IL-6, IL-1β, TNF-α, in the monocytes [10]. However, the specific mechanism triggering inflammation and injuries of adipoctyes has never been explored till now. Therefore, we discussed the Pg-LPS treated inflammatory status of the adipoctyes (illustrating by IL-6 and TNF-α) and explored the mechanism. Our findings showed that the Pg-LPS treatment significantly induced the increased levels of IL-6 and TNF-α, which are consistent with the previous study [22].

The functions of XBP1 were also clarified in the adipocytes in this study. Our results indicated that the XBP1 over-expression significantly enhanced the IL-6 and TNF-α levels in adipocytes. Meanwhile, XBP1 treatment also aggravated Pg-LPS stimuli caused inflammatory responses. However, the XBP1 silence significantly weakened the Pg-LPS stimuli induced inflammatory responses. These results suggest that the XBP1 over-expression enhances inflammation and XBP1 silence reduces the Pg-LPS-induced inflammation, which are consistent with the previous report [23]. However, a previous study [24] showed that the XBP1 could relieve the endoplasmic reticulum stress and alleviate the inflammation. Therefore, the expression of XBP1 plays critical roles in the inflammation of adipocytes and the XBP1 silencing could be considered as a potential therapeutic target for inflammation responses in adipocytes.

In this study, the adipocyte-derived molecules, adiponectin and leptin [25], were also examined in Pg-LPS and/or XBP1 (or XBP1 RNAi) treated adipocytes. For the gene transcription of the adiponectin and leptin molecule, there are significant differences between XBP1 silencing (pLVX-XBP1-RNAi) and XBP1 over-expression (pLVX-XBP1) group. According to a previous study [26], the leptin could modulate the nutritional status, inhibit fat synthesis, promote fat decomposition, therefore, leptin could reflect the status of adipocytes and fat accumulation in Pg-LPS treated adipocytes. Our results showed that the XBP1 over-expression significantly increased and XBP1 silence significantly decreased the leptin expression both in adipocytes stimulated with and without the Pg-LPS. These results suggest that the XBP1 over-expression inhibits fat synthesis and XBP1 silencing inhibits the fat decomposition. Adiponectin is mainly characterized by remarkable anti-atherogenic, anti-inflammatory and insulin-sensitive features [27,28]. The XBP1 over-expression significantly reduced and XBP1 silence significantly enhanced adiponectin expression in adipocytes undergoing treatment with and without Pg-LPS. These results hint that XBP1 over-expression in-directly induces inflammation and XBP1 silence in-directly inhibits inflammation. Therefore, XBP1 plays critical roles in modulating the inflammatory response and fat metabolism of adipocytes.

We also investigated the effects of Pg-LPS and/or XBP1 expression on the apoptosis in the adipocytes by evaluating the expression of Bax and caspase 3. The Bcl-2 and Bax protein are the key molecules for the mitochondria-mediated apoptosis [29]. The caspase 3 is the down-stream molecule for directing inducing the cell apoptosis [30]. Our results indicated that XBP1 over-expression significantly decreased Bcl-2 expression and increased Bax/caspase 3 expression in both Pg-LPS treated with and without adipocytes. These results suggest that Pg-LPS induced apoptosis of adipocytes by enhancing expression of XBP1 and modulating Bcl-2/Bax signalling pathway molecules. Our finding demonstrated new mechanism for the Pg-LPS caused inflammation and apoptosis compared to the previous studies [5,30] and discovered the endoplasmic reticulum (ER)-mediated apoptosis of adipocytes for the first time.

Although a few interesting findings were discovered, there are also some limitations. Firstly, for the identification of differentiated adipocytes, the Oil Red O staining method was used in this study. However, the adipocytes have not been identified with more optimal methods, such as immunocytochemistry (staining aP2 molecule, S-100 molecule) and adipogenic differentiation method. Secondarily, the protein contents of inflammatory factors and cytokines secreted by adipocytes have not been detected (such as using ELISA), only mRNA levels were determined.

In conclusion, we discovered that the Pg-LPS induced expression of inflammatory factors (IL-6, TNF-α and adiponectin) and activated apoptosis-associated molecule (caspase 3) in adipocytes, which are closely associated with the over-expression of XBP1. Therefore, the XBP1 over-expression induced the inflammation of adipocytes through activating IL-6, TNF-α, leptin and reducing the adiponectin expression. XBP1 over-expression triggered the apoptosis of adipocytes by initiating the mitochondria-mediated apoptosis. Therefore, in summary, *Porphyromonas gingivalis* lipopolysaccharide (Pg-LPS) induced adipocytes injuries through modulating XBP1 expression and initialling mitochondria-mediated apoptosis

## Materials and methods

### Adipocytes isolation and primary culture

The pre-adipocytes were isolated from the specific pathogen-free (SPF) and 4 weeks old SD rats purchasing from the Experimental Animal Centre of Chongqing Medical University (Chongqing, China). The isolation of pre-adipocytes were conducted using collagenase digestion approach according to the previous study described [31]. Briefly, the digested adipose tissues were filtered using the nylon screen (30 μm, Miltenyi BioTech., Aubum, CA, USA). The isolated pre-adipocytes were harvested by centrifuging for 10 min (200 ×), seeded onto culture plates and cultured in differentiation medium consisting with DMEM (Gibco BRL. Co. Ltd., Grand Island, New York, USA), 10% foetal bovine serum (FBS, Gibco BRL. Co. Ltd.) and 20 nM insulin (Sigma-Aldrich, St. Louis, Missouri, USA) and 3,3ʹ,5-triiodo-1-thyronine (Sigma-Aldrich). For the induction of the mature adipocytes, the pre-adipocytes were cultured in the differentiation medium supplementing with 0.125 M indomethacin, 0.5 M dexamethasone and 0.5 M isobutylmethylxanthine, all of which were purchased from Sigma-Aldrich. Post the induction, the produced cells were cultured in differentiation medium for 3–4 days, until cells illustrating accumulation of lipid droplets.

### Adipocytes culture and Pg-LPS treatment

The adipocytes were cultured with 5% CO2, at 37 °C in the DMEM (Gibco BRL.), supplementing with 10% FBS (Gibco BRL.). Adipocytes were seeded at the density of 5 × 10^5^ cells/ml medium in different cell culturing plates. For treatment with Pg-LPS, the culture medium was replaced with the complete-medium containing Pg-LPS (Carlsbad, CA, USA) at 100 ng/ml and incubated for 4 h, 8 h, 12 h and 24 h. The adipocytes were collected for quantifying the gene transcription and protein expression of cytokines or inflammatory factors.

### Oil Red O staining

The adipose tissues were fixed using the 4% paraformaldehyde (Beyotime Biotech. Shanghai, China) for 10 min and imbedded in O.C.T. Then, the adipose tissues were frozen and cut into sections, stained using the 3% Oil Red O (Cat. No. D027-1-1, Nanjing Jiancheng Bioengineering Institute, Nanjing, China) for 10 min, as the previous study described [32].

### Establishment of lentiviral vectors carrying XBP1 mimic and XBP1 RNAi and lentivirus packaging

**Figure 6.**
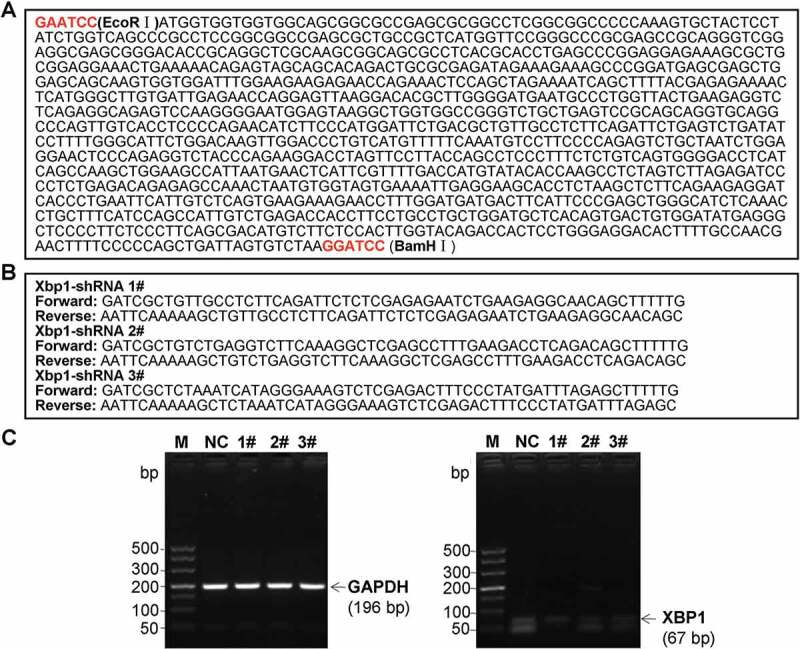
Oligonucleotide sequences for XBP1 mimic (a) and XBP1 RNAi (b), and identification for the silence efficacy of XBP1 RNAi (c)

The pLVX-IRES-ZsGreen1 lentiviral vector (pLVX, Clontech. Laboratories Inc., Palo Alto, CA, USA) was utilized to structured pLVX-XBP1-IRES-ZsGreen1 (pLVX-XBP1) plasmid and pLVX-XBP1-RNAi-IRES-ZsGreen1 (pLVX-XBP1-RNAi) plasmid. Meanwhile, the negative sham oligonucleotides for the XBP1 (pLVX-NC) were also synthesized by Western BioTech. (Shanghai, China). Oligonucleotide sequences for XBP1 mimic (Figure 6(a)) and XBP1 RNAi (Figure 6(b,c), 3 candidate sequences) were used to construct pLVX-XBP1 and pLVX-XBP1-RNAi, respectively. Then, the above oligonucleotide sequences were sub-cloned into the pLVX-IRES-ZsGreen1 lentiviral vector to construct pLVX-XBP1, pLVX-XBP1-RNAi and pLVX-NC plasmids.

Twenty-four hours prior to transfection, 293 T cells were cultured in a 10-cm dish. The pLVX-XBP1, pLVX-XBP1-RNAi and pLVX-NC and packing plasmids, including psPAX2, pMD2.G, were transfected with NDE3000TM Nanopolymer Transfection Reagent (Cat. No. WT1409-01, Western BioTech., Chongqing, China), as instructed by the manufacturer. About 72 h post-transfection, supernatants were collected by centrifuging (2200 × g) for 5 min at 4 °C. The collected supernatants were passed using a 0.45 μm syringe filter, and also cleared by centrifuging (20,000 × g) for 60 min at 4 °C. Titre of the isolated viruses were assessed by determining expression of ZsGreen based on instruction of manufacturer. Then, the above-synthesized lentivirus were transformed into *Escherichia Coli* competent cells DH5α (Cat. No. D9057, Takara, Tokyo, Japan). DH5α cells were subsequently inoculated to agarose medium to identify positive clones using enzyme digestions.

### ELISA

The adipocytes were lysed using RIPA Lysis Buffer (Cat. No. P0013 C, Beyotime Biotech. Shanghai, China) to obtain the XBP1 protein in cells. The XBP1 levels in adipocytes was measured with the commercial XBP1 ELISA detection Kit as described by instruction of manufacturer.

### Quantitative real-time PCR (qRT-PCR)

Total RNAs of adipocytes were extracted with TRIzol kits (Beyotime Biotech, Co. Int, Beijing, China) as manufacturer’s instruction described. The obtained total RNAs were reversely transcripted to generate complementary DNA (cDNA) using RNA Transcription Kit (Western Biotech. Chongqing, China). The obtained cDNAs were utilized for conducting the qRT-PCR assay. The PCR primers for were generated by Western Biotech. (Chongqing, China) and the associated sequences are listed in Table 1. SYBR Green I Real-Time PCR amplification system (Western Biotech. Chongqing, China) was employed to amplify the above genes as the described instruction by manufacturer. qRT-PCR were conducted on a Real-Time Fluorescence PCR instrument (Mode: FTC-3000P, Funglyn BioTech. Inc., Toronto, Canada) and the conditions were listed as the followings: 94 °C, 4 min, followed by 35 cycles of 94 °C, 20 s, 60 °C, 30 s, 72 °C, 30 s, and terminated at 72 °C for 10 min. All the above PCR reactions were conducted at least for 3 repeats. The 2^−ΔCt^ (2^−[(Ct of gene) − (Ct of U6)]^) approach was used to analyse and calculate the qRT-PCR findings [33].

**Table 1.** Primers for the qRT-PCR assay

Genes	Sequences	Length
XBP1		
Forward	GAAAGAAAGCCCGGATGAGC	152 bp
Reverse	ATTCATCCCCAAGCGTGTCC	
TNF-α		
Forward	GCCACCACGCTCTTCTGTC	149 bp
Reverse	GCTACGGGCTTGTCACTCG	
Leptin		
Forward	CACCCCATTCTGAGTTTGTCC	118 bp
Reverse	CTCGCAGGTTCTCCAGGTC	
Adiponectin		
Forward	TGTTCTTGGTCCTAAGGGTGAC	138 bp
Reverse	CCTACGCTGAATGCTGAGTGA	
β-actin		
Forward	CCCATCTATGAGGGTTACGC	150 bp
Reverse	TTTAATGTCACGCACGATTTC	
GAPDH		
Forward	AGTGCCAGCCTCGTCTCATA	97 bp
Reverse	GAGAAGGCAGCCCTGGTAAC	

### Western blot assay

The adipocytes were lysed with RIPA Lysis Buffer (Cat. No. P0013 C, Beyotime Biotech. Shanghai, China) as instructed by protocol of manufacturer. The lysates were separated with 15% sodium dodecylsulfate polyacrylamide gel electrophoresis (SDS-PAGE, Amresco Inc., Solon, OH, USA), and electro-transferred onto PVDF (Millipore, Boston, MA, USA). PVDF membranes were blocked with 5% defatted milk in PBS containing 0.05% Tween-20 (Beyotime Biotech, Co. Int, Beijing, China), with pH 7.5. Then, the PVDF membranes were incubated using rabbit anti-rat Bax polyclonal antibody (1: 2000; Cat. No. ab53154, Abcam Biotech., Cambridge, Massachusetts, USA), rabbit anti-rat caspase 3 polyclonal antibody (1: 2000; Ca. No. ab4051, Abcam Biotech.) and rabbit anti-rat GAPDH polyclonal antibody (1: 2000, Cat. No. ab9485, Abcam Biotech.) at 4 °C overnight. Subsequently, the PVDF membranes were treated using 1:3000 horseradish peroxidase (HRP)-conjugated goat anti-rabbit IgG (Cat. No. AQ132P, Sigma-Aldrich, St. Louis, Missouri, USA). Western blot bands were visualized using BeyoECL Plus kit (Cat. No. P0018S, Beyotime Biotech. Shanghai, China). Eventually, western blot bands were imaged and analysed using LabworksTM Analysis Software (version: 4.6, Labworks, Upland, CA, USA).

### Statistical analysis

Data were analysed using professional SPSS software (version: 20.0, IBM Corp., Armonk, NY, USA). The continuous variables were represented as mean±SD. Tukey’s post hoc test was used to validate ANOVA test for comparing continuous data among multiple groups. A statistical significance was defined when *p* < 0.05. All the experiments or the tests were conducted at least for 6 repeats.
